# Generalized low levels of serum N‐glycans associate with better health status

**DOI:** 10.1111/acel.13855

**Published:** 2023-05-02

**Authors:** Jiteng Fan, Jichen Sha, Shuwai Chang, Huijuan Zhao, Xiaoyun Niu, Yong Gu, Jianxin Gu, Shifang Ren

**Affiliations:** ^1^ NHC Key Laboratory of Glycoconjugates Research, Department of Biochemistry and Molecular Biology, School of Basic Medical Sciences Fudan University Shanghai China

**Keywords:** caloric restriction, liver, N‐glycans, O‐acetylation, serum, transcriptome

## Abstract

Caloric restriction (CR) can prolong life and ameliorate age‐related diseases; thus, its molecular basis might provide new insights for finding biomarker and intervention for aging and age‐related disease. Glycosylation is an important post‐translational modification, which can timely reflect the changes of intracellular state. Serum N‐glycosylation was found changed with aging in humans and mice. CR is widely accepted as an effective anti‐aging intervention in mice and could affect mouse serum fucosylated N‐glycans. However, the effect of CR on the level of global N‐glycans remains unknown. In order to explore whether CR affect the level of global N‐glycans, we performed a comprehensive serum glycome profiling in mice of 30% calorie restriction group and ad libitum group at 7 time points across 60 weeks by MALDI‐TOF‐MS. At each time point, the majority of glycans, including galactosylated and high mannose glycans, showed a consistent low level in CR group. Interestingly, O‐acetylated sialoglycans presented an upward change different from other derived traits, which is mainly reflected in two biantennary α2,6‐linked sialoglycans (H5N4Ge2Ac1, H5N4Ge2Ac2). Liver transcriptome analysis further revealed a decreased transcriptional level of genes involved in N‐glycan biosynthesis while increased level of acetyl‐CoA production. This finding is consistent with changes in serum N‐glycans and O‐acetylated sialic acids. Therefore, we provided one possible molecular basis for the beneficial effect of CR from N‐glycosylation perspective.

## Graphical abstract

Caloric restriction induced a general low level of serum N‐glycans in C57BL/6 mice across 60 weeks, and genes and pathways involved in glycosylation synthesis were also down‐regulated in CR group. A combination of glycome and transcriptome analysis suggested that caloric restriction affects the glycosylation mechanism, which is expected to be a marker of better health status.

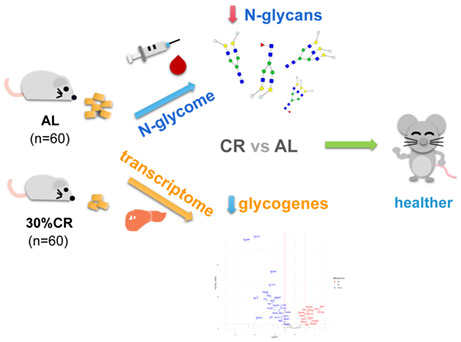

**Abbreviations**
Ac: O‐Acetylation of N‐glycolylneuraminic acid
ACN: Acetonitrile
CR: calorie restriction
DMSO: dimethyl sulfoxide
E: ethyl esterified N‐acetylneuraminic acid (α2,6‐linked)
EtOH: ethanol
F: fucose
FA: formic acid
Ge: ethyl esterified N‐glycolylneuraminic acid (α2,6‐linked)
GEO: gene expression omnibus
Gl: lactonized N‐glycolylneuraminic acid (α2,3‐linked)
GO: Gene Ontology
GSEA: gene set enrichment analysis
H: hexose
HOBt: 1‐hydroxybenzotriazole monohydrate
KEGG: Kyoto Encyclopedia of Genes and Genomes
L: lactonized N‐acetylneuraminic acid (α2,3‐linked)
MALDI‐TOF‐MS: Matrix‐Assisted Laser Desorption/Ionization Time of Flight Mass Spectrometry
MQ: Milli‐Q water
N: HexNAc
NaBD4: sodium borodeuteride
NaOH: sodium hydroxide
NP‐40: Nonidet P 40
PBS: phosphate buffered solution
PNGase F: peptide‐N‐glycosidase F
SDS: sodium dodecyl sulfate
SIAE: sialic acid esterase
Siglecs: sialic‐acid‐binding immunoglobulin‐like lectins
SOAT: sialic acid‐specific O‐acetyltransferase
super‐DHB: super‐2,5‐dihydroxybenzoic acid
TFA: trifluoroacetic acid

## 1 INTRODUCTION

Calorie restriction (CR), the long‐term reduction in calorie intake without causing malnutrition, is the most effective way to extend life by far. CR extends the healthy life span of varies organisms, including yeast, worms, rodents, and primates (Mattison et al., 2017; Weindruch, 1996). Meanwhile, as the most effective anti‐aging intervention, CR can delay or suppress age‐related biological changes and chronic metabolic diseases, including diabetes, cardiovascular diseases even cancers (Colman et al., 2009; Kim et al., 2020; Napoleao et al., 2021). CR also plays an effective anti‐inflammatory role in different diseases (Procaccini et al., 2023). Many studies have shown the positive effects of CR on health, both after short and long periods of CR, resulting in weight loss and improvement of physiological markers (Ma et al., 2020; Most et al., 2017). Therefore, understanding molecular basis might provide new insights for finding biomarker and intervention for aging and age‐related diseases.

Glycosylation, as one of most important and universal of post‐translational modification, is not controlled by template coding like protein, but is concurrently influenced by glycosyltransferases and glycosidases, nucleotide sugars, and transporters. Therefore, the spatial and temporal changes of glycosylation are more likely to reflect the real time state of cells (Haltiwanger & Lowe, 2004; Knezevic et al., 2010; Ohtsubo & Marth, 2006). The cellular physiological environment undergoes complex changes during aging and disease. Earlier studies found regular changes in serum N‐glycans with aging. For example, three N‐glycan structures (NGA2F, NA2FB, and NA2F) were observed to be age‐related, and an age‐dependent rise in the relative abundance of agalactosylated glycans was observed in both males and females (Vanhooren et al., 2007, 2009). In addition, plasma glycosyltransferase levels also showed an age‐related changing (Catera et al., 2016). Meanwhile, the alterations in protein glycosylation profiles were also reported associated with age‐related diseases, such as cancer and metabolic syndrome (Rudman et al., 2019; Silsirivanit, 2019). In recent report, after 8 weeks on a low‐calorie diet (LCD), the obese subjects showed a shift in their IgG glycotypes from pro‐inflammatory to anti‐inflammatory activity (Deris et al., 2022). Moreover, in mice serum, CR, as an effective anti‐aging intervention in mammals, has been reported that can affect age‐related fucosylated glycans change in mice (Vanhooren et al., 2011). However, the effect of CR on the level of global N‐glycans remains unknown because only few serum glycans after removing sialic acid residues were detected previously.

Although serum N‐glycans analysis after desialylation can effectively analyze fucosylated glycan changes, profiling the global N‐glycans retaining the sialic acid residues is very important. One reason is that desialylation makes it difficult to determine whether some neutral glycans detected are produced after the removal of sialic acid. Another reason is the loss of sialic acid information. Sialylated glycans has been reported to be associated with aging and age‐related diseases including inflammation and cancer (Taniguchi & Kizuka, 2015; Zhang et al., 2018). For instance, elevated sialylated is a common feature of various tumor cells, and changes in serum sialylation are associated with tumor transformation and disease progression (Berbec et al., 1999; Wongkham et al., 2001; Zhang et al., 2018). It was found that sialic acid structures can specifically bind to sialic‐acid‐binding immunoglobulin‐like lectins (siglecs) which are inhibitory receptors on immune cells, and regulate immune cell responses and cancer progression (Hudak et al., 2014; Schwarz et al., 2015). What's more, sialic acid is a special monosaccharide of N‐glycan with a negative charge in the cell environment. There may be functional group modifications on sialic acid at the C‐4, C‐7, C‐8, and C‐9 hydroxyl groups, among which O‐acetylation is widely found (Schauer, 1970a, 1970b). In the Golgi, SOAT can add acetyl groups provided by acetyl‐CoA to CMP‐sialic acid, and deacetylation of O‐acetylated sialic acid is mediated by SIAE (Mandal et al., 2015; Visser et al., 2021). O‐acetylation increases the hydrophobicity of sialic acid and has an effect on antigen and lectin recognition. For example, O‐acetylation reduces the binding ability of siglec‐2 on the surface B cells to sialic acid, which may be a molecular switch for signaling (Cariappa et al., 2009; Sjoberg et al., 1994).

In this study, we investigated the effect of CR on serum N‐glycans globally including all of original N‐glycans released from serum glycoproteins. We subjected C57BL/6 mice of both genders to caloric restriction and quantified serum N‐glycome at seven time points (15w, 19w, 23w, 27w, 31w, 35w, 60w). In addition, our method is different from previous quantitative methods, which quantified and normalized each glycan by calculating its peak intensity to the total signal intensities of all glycans in one sample. On the contrary, in the Bionic Glycome Quantitation method we employed (Qin et al., 2019), each glycan has its own internal standard and thus the level of each glycan can be quantified individually in the sample with CR treatment or not. Finally, 58 glycans were identified by this method, of which 45 glycans can be quantified. The majority of glycans, including galactosylated and high mannose glycans, showed a consistent low level in CR group across all the seven time points. Interestingly, the minority of O‐acetylated glycans which only had α2,6‐linked sialic acid showed a consistent high level in CR group. Considering the reported masking effect of O‐acetylation on terminal sialic acid (Cariappa et al., 2009; Sjoberg et al., 1994), the low level of serum N‐glycans may contribute to the health beneficial effect of calorie restriction. Liver transcriptome analysis further revealed significant changes in N‐glycan synthesis genes and pathways, confirming the possible important role of N‐glycans in the process of calorie restriction and the great potential for N‐glycans to develop into biomarkers.

## 2 MATERIALS AND METHODS

### 2.1 Animals and reagents

C57BL/6 mice of 8 weeks years old (*n* = 120) were purchased from Shanghai jiesijie experimental animal Co., Ltd. All animal experiments were in accordance with the requirements of the Animal Experiment Ethics Committee of Fudan University and were approved. Animals were maintained on commercial normal diet (proteins 24.02%, fat 12.95%, carbohydrates 63.03%, 3.44 kcal/g) from Beijing Keao Xieli Feed Co., Ltd. Complete diet composition is listed in Table S1. The environment was kept at 23 ± 2°C, and 12 h light/dark cycle with light on at 06:00 a.m.

*Reagents*: Sodium dodecyl sulfate (SDS), 1‐hydroxybenzotriazole monohydrate (HOBt), sodium borodeuteride (NaBD_4_), formic acid (FA), trifluoroacetic acid (TFA), sodium hydroxide (NaOH), super‐2,5‐dihydroxybenzoic acid (super‐DHB), 10 × phosphate buffered solution (PBS), dimethyl sulfoxide (DMSO) and Sepharose CL‐4B were purchased from Sigma Aldrich. Acetonitrile (ACN) and ethanol (EtOH) were provided by Merck Millipore. NP‐40 and peptide‐N‐glycosidase F (PNGase F) were from New England Biolabs (Inc.). The FiltrEX™ 96‐well Clear Filter Plates with 0.2 μm PVDF Membrane were from Corning. Milli‐Q water (MQ) was provided by Milli‐Q system.

### 2.2 Diets and samples collection

Prior to the diet intervention, the food was weighed at the same time every day for 2 weeks, and the average of the reduced food intake was used as the normal intake. At the start of the diet, 12‐week‐old animals were randomly divided into two groups, AL (ad libitum) group (30 male and 30 female) and CR group (30 male and 30 female). AL group were maintained in groups of five animals; each mouse of CR group was kept in a separate cage to prevent food competition. CR mice were fed once per day 2 h after the lights turned off, and food was placed onto the floor of cage. AL group were fed in the hopper. CR mice get 10 percent less per week until they reached 30 percent calorie restriction, while AL group receiving normal diet ad libitum. All mice were provided with ad libitum access to water. All of the mice were weighed every 2 weeks.

Blood samples were collected from the eye socket of mice by using capillary tubes without sacrificing the mice. Serum samples were collected on 15w, 19w, 23w, 27w, 31w, and 35w between 14:30 and 16:00 p.m. Samples were centrifuged at 3000 rpm for 10 min after 3 h quiescence. The serum was separated and stored at −80 degrees. Serum samples had not been frozen and thawed more than three times.

After 18 weeks of CR diet, 30‐week‐old male mice (*n*[AL] = 3 and *n*[CR] = 4) were euthanized by cervical dislocation between 14:30 and 16:00 p.m. Liver tissues were rapidly harvested and then flash‐frozen in liquid nitrogen and stored at −80°C. At 60 weeks old, all of the remaining mice were euthanized in the same way. Blood was obtained before sacrificed by enucleation of the eyeball, and serum was separated by the method described above. White adipose tissues (WAT) were collected and fixed in formalin.

### 2.3 Serum protein quantification and adipose tissue staining

According to the manufacturer's method, the serum of three time points (15w, 27w, 31w) were selected randomly for protein content quantification by Pierce™ BCA Protein Assay Kit (Thermo Fisher Scientific).

Formalin‐fixed WAT were embedded in paraffin (three mice per group). All the samples were cut into 5–6 μm paraffin sections and stained with hematoxylin and eosin (H&E). Finally, a Nikon light microscope (Nikon) was used to scanned for the entire field of view of paraffin sections, and images at 20× and 40× resolution were obtained.

### 2.4 N‐glycans release and Bionic Glycome preparation

As for N‐glycans release, 10 μL 2% SDS was added to 5 μL of serum and incubated at 60°C for 10 min. After denaturation, adding 10 μL enzymatic hydrolysis buffer (4% NP‐40 and 5 × PBS) and 1 μL PNGase F and the reaction was carried out overnight at 37°C.

The Bionic Glycome Method was used to make the internal standards (Qin et al., 2019). We selected mouse serum randomly to make a serum mix. The mix was precipitated with twice the volume of ice EtOH. The supernatant was transferred to a new centrifuge tube; 1% volume of FA was added and incubated at 37°C for 2 h. 2 M of freshly prepared NaBD_4_ solution was used for internal standard reduction and reacted at 60°C for 2 h. Moreover, we optimized the purification part from before. We used Sepharose beads instead of the HILIC SPE column of cotton to simplify the procedure without compromising the purification effect. This facilitates the preparation of internal standards in large quantities. The activated Sepharose CL‐4B beads were added to the 96‐well PVDF membrane, and the samples were added to the wells and incubated for 15 min. After centrifugation, the beads were washed with 200 μL of 95%ACN containing 1%TFA to fully remove impurities. Finally, the glycans were eluted with MQ and concentrated by vacuum centrifugation, dried and stored at −80°C.

### 2.5 Sialic acid derivatization and purification of N‐glycans

Two μL of released N‐glycans or internal standard (10 μL of MQ redissolved) was added with 20 μL of derivatization reagent (250 mM EDC and 250 mM HOBt dissolved in EtOH) and reacted for 1 h at 37°C. After cool down to room temperature, 22 μL of ‐ACN was added and the reaction was carried out at −20°C for 15 min. The precipitated proteins were removed, and the glycans were purified using HILIC‐SPE tips made of cotton as reported in previous studies (Reiding et al., 2014). Purified glycans finally eluted in 10 μL of MQ.

### 2.6 MALDI‐TOF‐MS analysis

Bruker ultrafleXtreme laser equipped with Smartbeam‐II was used to detect the purified glycans in a reflected positive ion mode combined with the support of proprietary software Flexcontrol 3.4 (Bruker Daltonics). Calibration was performed using the Bruker Peptide Calibration Standard II. Each purified sample was mixed with an equal volume of the internal standard and dot 1 μL on a MTP 384 target plate polished steel BC (Bruker Daltonics), with three duplicate dots per sample. After air‐dried, 1 μL of substrate (newly prepared 5 mg/mL super‐DHB, dissolved in 1 mM NaOH in 50% ACN) was added and allowed to dry at room temperature. The range of m/z ratio was set from 700 to 3500. Laser emission was set at 10,000 laser shots and strafed samples with 100 shots per grating spot by complete random walk at a frequency of 1000 Hz; the laser voltage is set at 68 V. Glycans structures were confirmed by tandem mass spectrometry (MALDI‐TOF‐MS/MS), and GlycoWorkbench (version 2.1) (https://code.google.com/p/glycoworkbench/) was used for fragmentation spectra analysis.

### 2.7 Data processing and statistical analysis

The mass spectrograms were analyzed using flexAnalysis (Bruker Daltonics). Through the known m/z ratio (1647.5865 (H4N4F1), 1836.65066 (H4N4Ge1), 2479.88342 (H5N4F1Ge2), 1998.70346 (H5N4Ge1), 2287.77848 (H5N4Ge1Gl1), 2333.82552 (H5N4Ge2), 2622.90054 (H5N4Ge2Gl1)), the instrument was calibrated to reduce mass drift between sample batches. A total of 58 pairs of glycans were identified (S/N > 3). The data processed by flexAnalysis software were further imported into the commercial BioPharma Compass software (Bruker Daltonics) to extract peak signal intensity of each N‐glycan spectrum. The relative peak signal intensity (light/heavy peak intensity) of each pair of target peaks was used for quantification, and the average signal intensity of three repeats was included in the quantitative analysis. A total of 58 glycans were identified, of which 45 glycans with coefficient of variation (CV) less than 25% in the reproducibility test were selected for quantification, and 18 derived glycosylation traits were further calculated (Tables S2 and S3). The formula used is shown in Table S4.

To exclude the effect of outliers, we removed outliers larger than Q3 + (1.5) × IQR or smaller than Q1 − (1.5) × IQR, where IQR = Q3 − Q1 (Q1 is 25% quantile and Q3 is 75% quantile). Statistical analysis was performed by IBM SPSS Statistics 20 (IBM Corporation), GraphPad Prism7.0 software (GraphPad Software Inc.), and R 4.1.0.(R Foundation for Statistical Computing) were used to create the diagrams. The difference between glycans was compared using an unpaired Student's *t*‐test. *p < 0.05; **p < 0.01; ***p < 0.001; ****p < 0.0001.

### 2.8 Validation by isotope‐labeled permethylation method

To validate our results, we designed an isotope‐labeled permethylation derivatization method for the quantification of glycans. The samples were subjected to methylation derivatization as shown previously (Blomme et al., 2011). Briefly, NaOH pellets and DMSO were grind thoroughly, and 0.2 mL was added to the dried sample purified by Sepharose. Add 0.2 mL of iodomethane, shake thoroughly for 1 h and then add water to quench the reaction. Add chloroform to extract the glycans and remove the aqueous phase. Multiple additions of water were used for extraction to achieve purification. In addition, the mixed serum was purified and derivatized with deuterated iodomethane, and the rest of the processing was the same. After spin‐drying and re‐dissolving with 50% methanol, the sample and the internal standard were mixed for MALDI‐TOF‐MS analysis.

### 2.9 Liver transcriptome analysis

Liver tissues were used for transcriptome analysis. Genome‐wide gene expression profiling with 150 bp paired‐end reads length was performed on Illumina platform. Briefly, mRNA was purified from total RNA using poly‐T oligo‐attached magnetic beads. cDNA library constructed by reverse transcription synthesis and was detected by Agilent 2100 bioanalyzer to conform the integrity and total amount. According to the manufacturer's instructions, the clustering of the index‐coded samples was done, and the library preparations were sequenced on an Illumina Novaseq platform and 150 bp paired‐end reads were produced.

Differential expression analysis was performed by the DESeq2R package (1.20.0). To control the false discovery rate, the obtained *p*‐values were adjusted by Benjamini and Hochberg's method. Gene Ontology (GO) enrichment analysis of differently expressed genes and statistical enrichment in Kyoto Encyclopedia of Genes and Genomes (KEGG) pathways were implemented by the cluster Profiler R package. Gene Set Enrichment Analysis (GSEA) is a computational method used to determine whether a given set of genes can show significant consistency differences between two biological states. We use the local version of the GSEA analysis tool (http://www.broadinstitute.org/gsea/index.jsp), and GO and KEGG were used for GSEA independently. The RNA‐seq data have been submitted to the Gene Expression Omnibus (GEO) database, and the accession number is GSE219147.

## 3 RESULTS

### 3.1 Calorie restriction caused a generalized downward in serum N‐glycome without protein decline

**FIGURE 1:**
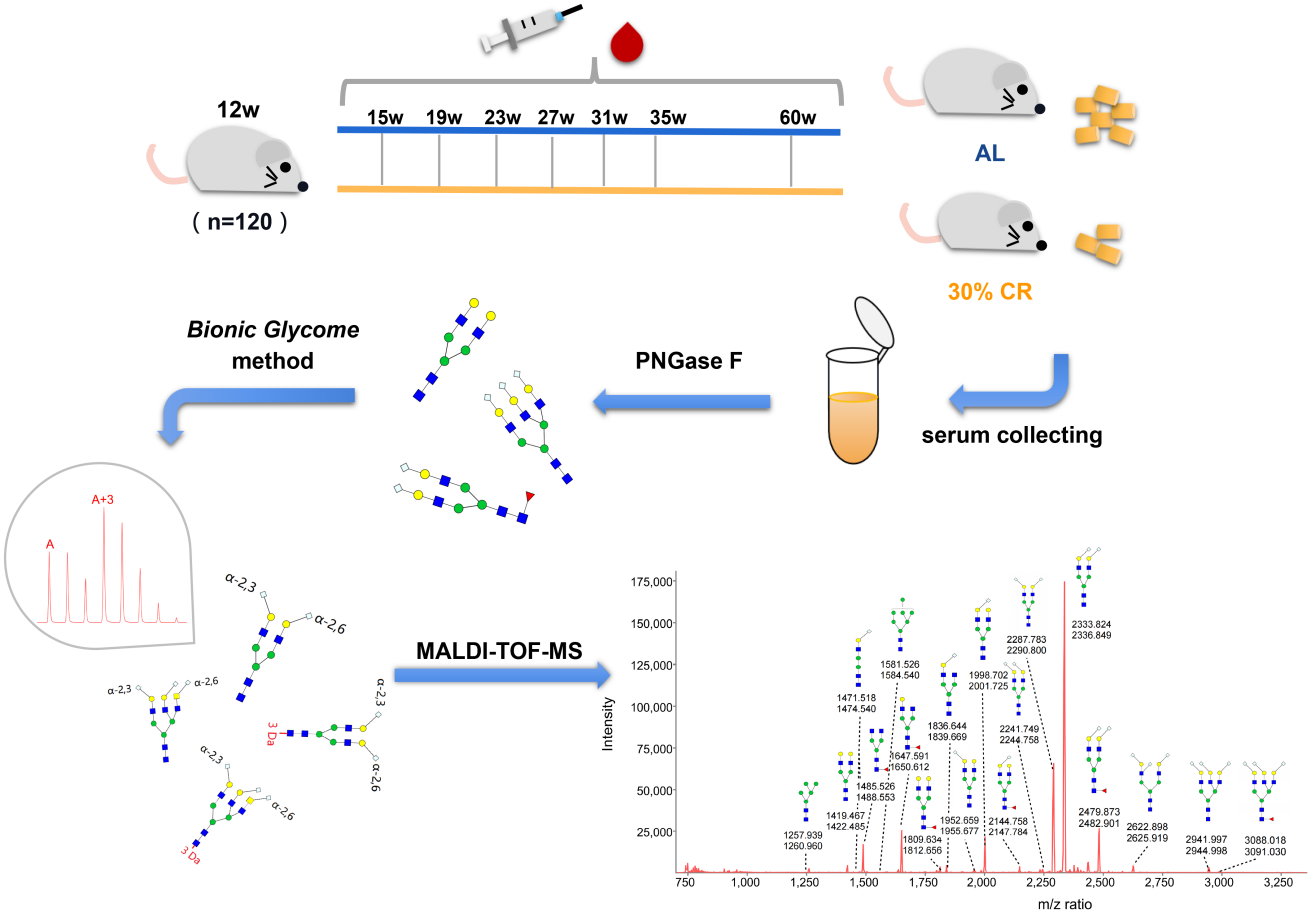
Experimental design for assessing the effect of calorie restriction on serum N‐glycans in mice. Bionic Glycome method provided an internal standard with 3 Da difference for each glycan to achieve accurate glycome quantitation. Structural symbols: blue square: N‐acetylglucosamine; green circle: mannose; red triangle: fucose; yellow circle: galactose; anticlockwise white diamond: α2,3‐linked N‐glycolylneuraminic acid; clockwise white diamond: α2,6‐linked N‐glycolylneuraminic acid.

The experimental design is illustrated in Figure 1. A total of 120 C57BL/6 mice of 12 weeks old were randomly divided into two groups and exposed to ad libitum (AL) and calorie restriction (CR) diet. Mice in CR group (*n* = 60) were kept in separate cages and reduced by 10% per week until they stabilized at 30% calorie restriction after 3 weeks. During this process, food was placed at the bottom of the cage for easy access, and no food was left. The AL group (*n* = 60) was free to eat every day. The daily food intake of CR mice was 2.6–3.0 g, and energy was 8.944–10.32 kcal.

**FIGURE 2:**
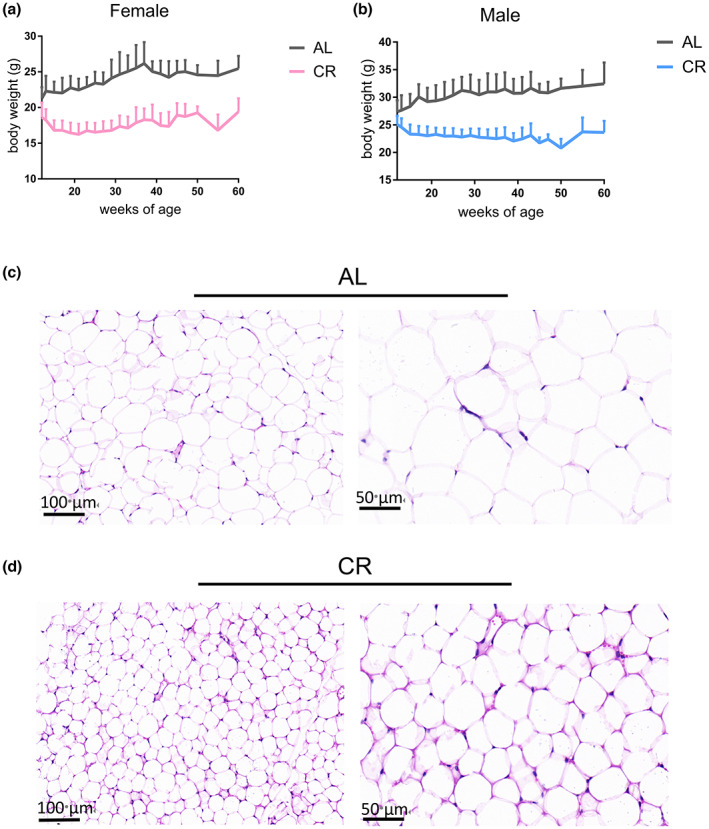
Physiological changes during calorie restriction. (a, b) Significant weight loss in both sex under CR. (c, d) Representative images of adipocyte size in AL and CR group at 60w in male mice. The size of adipocytes was significantly reduced after CR treatment.

In order to comprehensively analyze the dynamic changes in the serum N‐glycome of mice under long‐term CR, 562 serum samples were collected from the eye sockets. We selected several serum samples randomly to mix and prepared Bionic internal standards (IS), so as to better cover the total N‐glycome of serum proteins and avoid the lack of IS peaks. After derivatization and purification, internal standards and samples were mixed for MALDI‐TOF‐MS detection (Figure 1). Representative spectrogram was shown in Figure S1 (some glycans are not indicated). After caloric restriction, the body weight of both male and female mice decreased significantly, and gradually stabilized after reaching 30%CR (Figure 2a,b). Through the hematoxylin and eosin (H&E) stain of WAT, we found that the adipocyte size of WAT tissue of mice was significantly reduced after calorie restriction (Figure 2c,d).

Combining previous studies (Reiding et al., 2016; Rombouts et al., 2016) and tandem mass spectrum data, a total of 58 serum glycans were identified, of which 45 glycans were quantified (CV < 25%). To the best of our knowledge, this is the most N‐glycans identified in the mice serum of CR. The accuracy of our quantitation was further verified by the permethylation method. We compared the glycans identified by both methods, two derivatization methods yielded consistent results (Figure S2). However, the number of glycans identified was less due to the lack of recognition of sialic acid‐linked isomers by the permethylation method.

**FIGURE 3:**
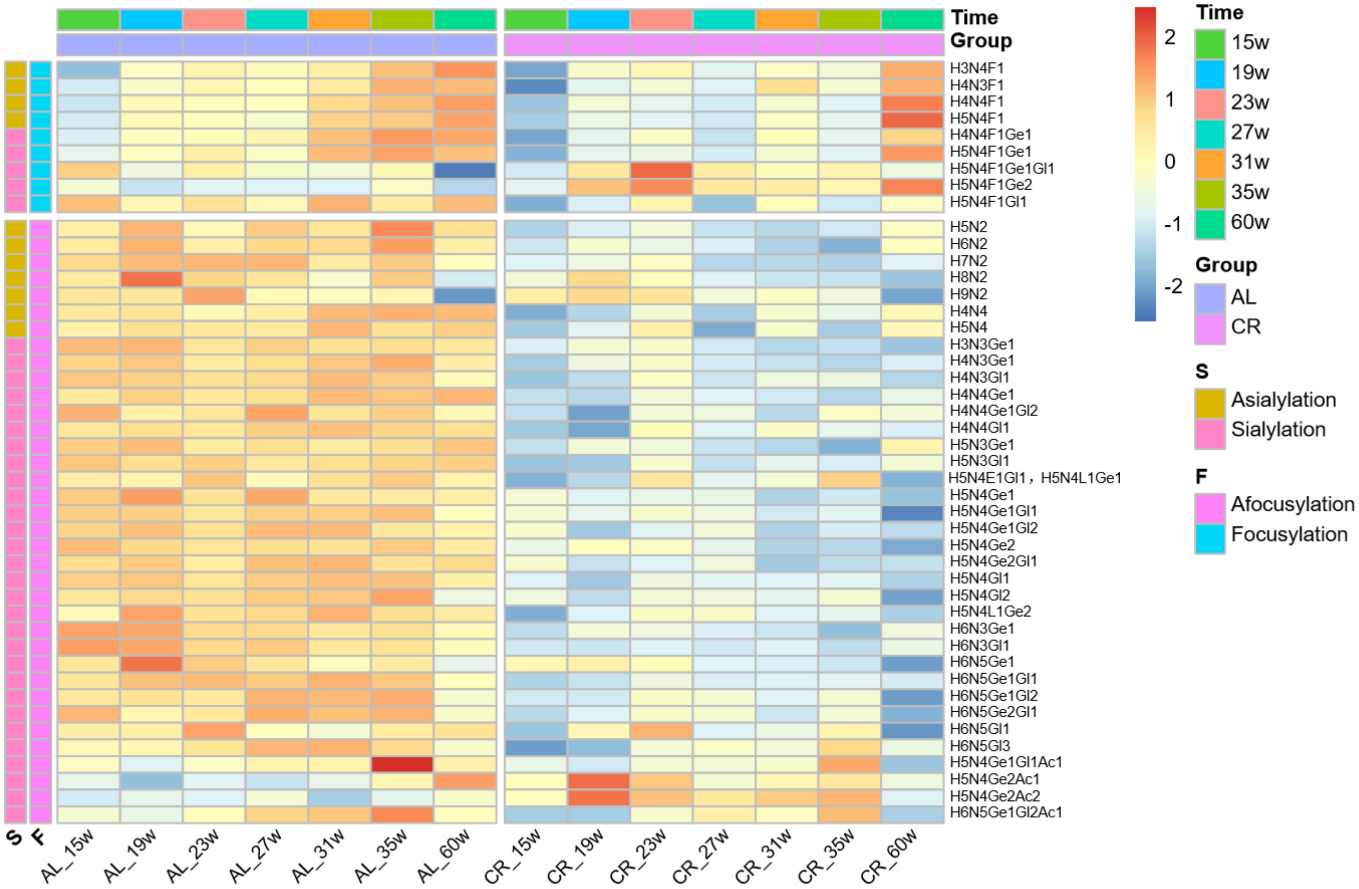
Heatmap of individual serum glycans change at seven time points (15w, 19w, 23w, 27w, 31w, 35w and 60w) in AL and CR groups. The value of each glycan in the heatmap is transformed by log_10_. A total of 45 glycans were quantitatively analyzed. Time, group, and derived glycans traits of S (asialylation and sialylation) and F (afocusylation and focusylation) were labeled on the side. O‐acetylated sialylated glycans were also identified, and majority of the glycans were decreased significantly in CR group. Structure abbreviations: Ac, O‐Acetylation of N‐glycolylneuraminic acid; E, ethyl esterified N‐acetylneuraminic acid (α2,6‐linked); F, fucose; Ge, ethyl esterified N‐glycolylneuraminic acid (α2,6‐linked); Gl, lactonized N‐glycolylneuraminic acid (α2,3‐linked); H, hexose; L, lactonized N‐acetylneuraminic acid (α2,3‐linked); N, HexNAc.

The change of each quantified N‐glycans during aging in AL and CR groups is shown in the heat map (Figure 3). As shown in the figure, compared with fucosylated glycans, afucosylated glycans were more abundant in the serum N‐glycans of mice. In addition, sialic acid linked to α2,3 and α2,6 can be distinguished in this study, and the sialic acid with O‐acetylation modification was also detected. The structures of 6 glycans were further identified by tandem mass spectrometry (Figure S3). Most of the identified glycans showed a significant and stable lower level at every time points in CR group. In order to elucidate whether this decrease was caused by changes in protein abundance, serum protein concentrations were assessed for three time points (Figure S4). We found that the amount of protein did not decrease and even increased slightly. Thus, calorie restriction caused a global decrease in serum N‐glycome levels without reducing serum protein levels.

### 3.2 Derived glycosylation traits changed during calorie restriction

**FIGURE 4:**
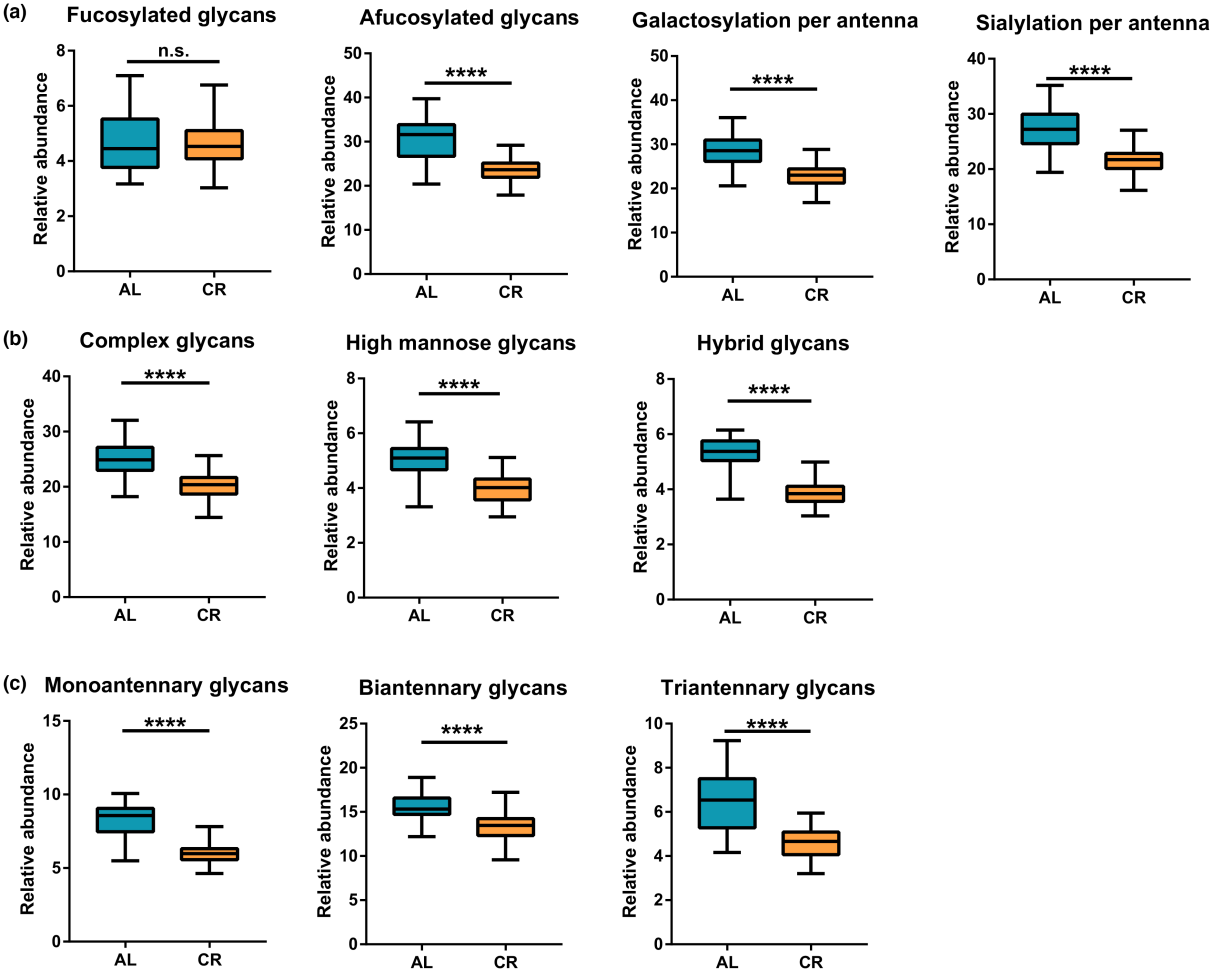
Relative abundance of derived glycosylation traits in AL and CR group. All serum samples from all seven time points were included in the analysis between glycans and CR. (a) According to the monosaccharides of glycans, four traits were divided into fucosylation, afucosylation, galactosylation per antenna, and sialylation per antenna. (b) As for structure, derived features included high mannose glycans, hybrid glycans and complex glycans. (c) Monoantennary, biantennary, and triantennary glycans were distinguished by antenna numbers. The difference between traits was compared using an unpaired Student's *t*‐test. The *p*‐value was considered significant if it was below 0.05. *p < 0.05; **p < 0.01; ***p < 0.001; ****p < 0.0001. “n.s.” stands for no significant difference.

In addition to analyzed and compared the total N‐glycome at the level of individual glycan structures, derived glycosylation traits were further assessed. We analyzed and compared the level of glycosylation traits derived based on structural similarity between AL and CR group (Figure 4 and Figure S5). It is found that the abundance of three types of glycans (high mannose, hybrid, and complex) was decreased obviously, and glycans with different antenna numbers also decreased in CR group. As for the derived glycosylation traits based on the component of monosaccharide, we found that the level of galactosylation and sialylation of per antenna remained low after CR, while fucosylation was not affected.

We further investigated whether gender difference has an effect on the alteration in derived glycan traits. Considering previous researches have reported that higher levels of core fucosylation were in females than in males in humans and mice (Ding et al., 2011; Han et al., 2020), we analyzed the effect of gender on the fucosylation. We found that the fucosylation level was significantly higher in female mice than that in male for both AL and CR groups (Figure S6a,b). Male had higher levels of afucosylated glycans. The result was consistent with previous findings. After CR treatment, the level of fucosylation trait showed opposite changing trend in male and female mice. Among the 9 fucosylated glycans we analyzed, the male of CR group increased mainly in the two biantennary sialoglycans (H5N4F1Ge1Gl1 and H5N4F1Ge2). Females in the CR group showed a significant decline in most glycans (Figure S6c–k). This suggested that fucosylation is significantly affected by sex, and its change with calorie restriction may be adjusted by sex. In contrast, when we analyzed the effect of gender on the other glycan traits, we found they tended to decrease in both sexes after CR, and interestingly, more in male (Figure S7). Therefore, we considered them as a group for further analysis.

### 3.3 N‐glycans with α2,3/α2,6‐linked sialic acid decreased while with O‐acetylated sialic acid interestingly elevated

**FIGURE 5:**
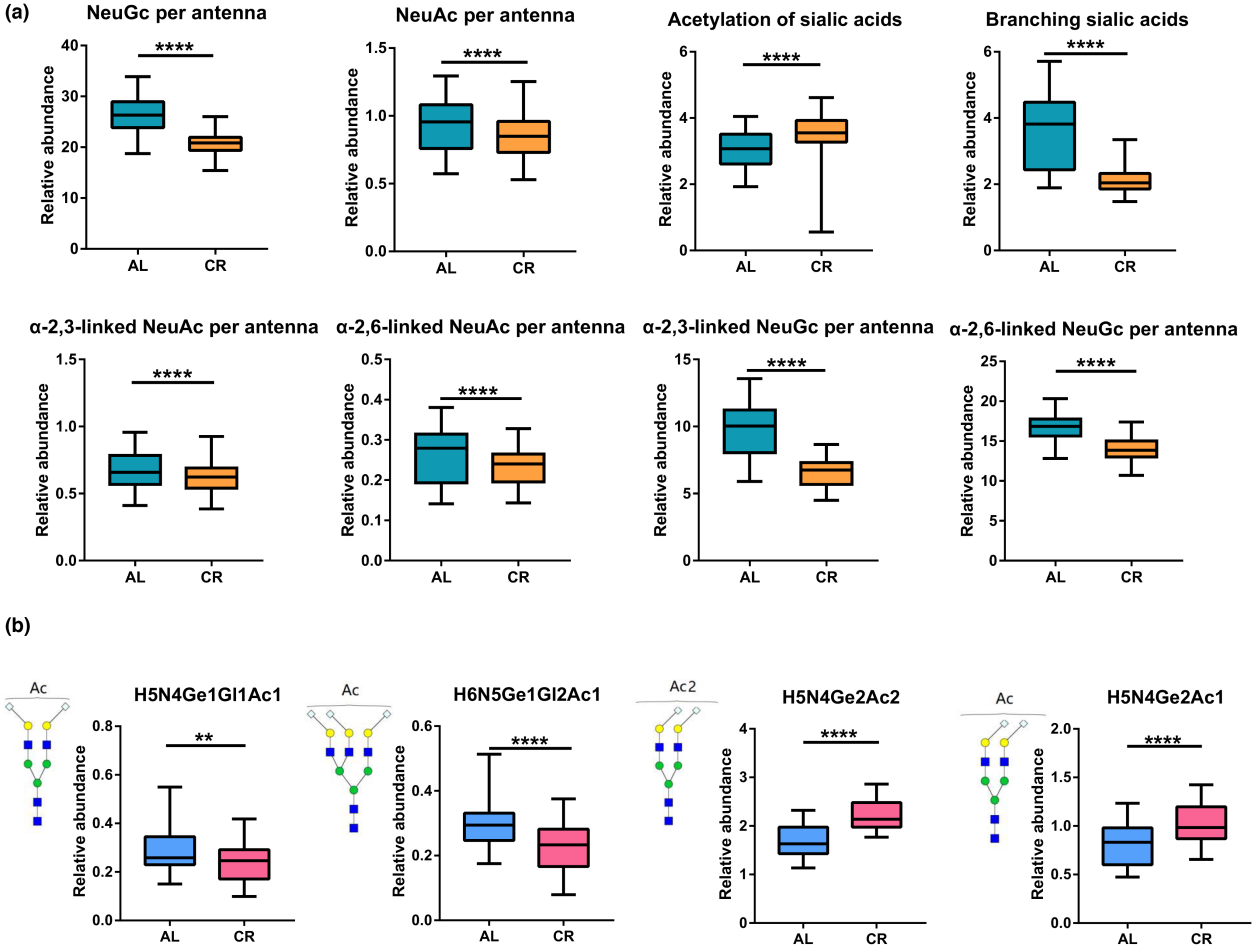
Relative abundance of derived traits of sialylation and O‐acetylated sialic acid glycans between AL and CR group. (a) Based on the link types and modification signatures, a total of 8 derived traits of sialylation were divided. “Branched sialic acid” is the glycans with disialylated antennae. (b) Changes of the glycans with O‐acetylated sialic acid during CR. Among the four acetylated sialoglycans that could be used for quantification, the two glycans only connected with α2,6 sialic acid (H5N4Ge2Ac1 and H5N4Ge2Ac2) increased significantly, H5N4Ge1Gl1Ac1 and H6N5Ge1Gl2Ac1 showed a decrease in the process of CR. *p < 0.05; **p < 0.01; ***p < 0.001; ****p < 0.0001.

Since the approach we employed allows detection and quantitation of individual N‐glycans with α2,3/α2,6‐linked sialic acid as well as O‐acetylated sialic acid, which are considered to play an important role in biological processes, we also evaluated the effect of CR on the properties of these sialic acid‐derived traits, as shown in Figure 5 and Figure S8. Neu5Gc, as the main form of sialic acid in mice serum, decreased in both linkage types after CR. At the same time, the sialoglycans in the form of Neu5Ac were less in the mice serum and also decreased in the CR group. Branched sialic acid showed the same change. Interestingly, the O‐acetylated sialic acid was significantly elevated. It is worth mentioning that acetylation of sialic acid has been reported to have the effect of masking sialic acid (Sjoberg et al., 1994; Visser et al., 2021).

Figure 5b shows the changes of the four O‐acetylated sialic acid glycans we identified under caloric restriction. We found that the abundance of H5N4Ge1Gl1Ac1 and H6N5Ge1Gl2Ac1 decreased after CR. However, the glycans containing only with α2,6‐linked sialic acid but not α2,3‐linked sialic acid modifications increased obviously during CR treatment. Previous studies have demonstrated 9‐O‐acetylation and the regulation of SIAE are related to the formation of α2,6‐linked sialic acid by ST6Gal1 (Grabenstein et al., 2021), suggesting a possible regulatory relationship between α2,6‐linked sialic acid and O‐acetylation in CR.

### 3.4 Liver transcriptome analysis showed that N‐glycan synthesis was significantly affected

Liver is the secretory organ of most serum proteins, participating in the body's energy metabolism, and also is responsible for maintaining metabolic homeostasis. To gain more insights into the regulation of N‐glycosylation under caloric restriction, liver transcriptome analysis for both AL and CR group was performed in this study. Samples within the group had a strong Pearson correlation (Figure S9a). Principal component analysis (PCA) of transcriptomic data (Figure S9b) indicated the significant differences between different feeding patterns. Venn diagram was used to show the differential expression of AL and CR groups (Figure S9c). There were 9969 shared transcripts, 614 specifically expressed transcripts in AL group and 513 specifically in CR group. The volcano plot showed that compared with the AL, 672 up‐regulated genes and 752 down‐regulated genes were found in CR (Figure S9d). Pathways and gene sets with noticeable changes were identified in enrichment analysis (Figure S10), among which processes related to xenobiotics and drug metabolism were up‐regulated, including xenobiotic metabolic, exogenous drug catabolic, drug catabolic, and cytochrome P450 pathway, and the uptrend of fatty acid metabolic activity was found, including long‐chain fatty acid metabolism, arachidonic acid metabolism, and unsaturated fatty acid metabolism process. In addition, the decreased of negative regulation of proteolysis and peptidase inhibitor activity were observed, which may further promote protein digestion.

**FIGURE 6:**
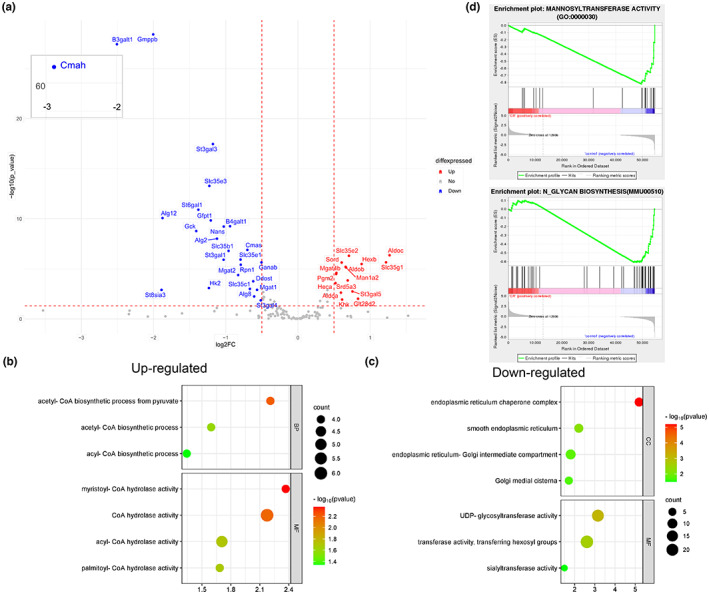
RNA‐Seq of mouse liver response to caloric restriction. (a) 148 glycogenes were analyzed; the genes with |log_2_Fc| > 0.5 and *p* < 0.05 were showed on the volcano plot. (b, c) The remarkable up‐regulated and down‐regulated enrichment (*p* < 0.05) in GO analysis related to N‐glycans biosynthesis. (d) Significant decreases were found in mannosyl transferase activity (GO:0000030) (*p* < 0.001) and N‐glycan biosynthesis (MMU00510) (*p* < 0.001) in GSEA of GO and KEGG analysis.

In order to understand the changes of genes and pathways related to N‐glycan synthesis, 148 key genes in nucleotide sugar synthesis pathway, nucleotide sugar transporters, glycosidases, and glycosyltransferases were analyzed. As shown in Figure 6a, 15 genes increase slightly, while 25 genes significantly decrease obviously (|log_2_Fc| > 0.5 and *p* < 0.05). Among them, N‐acetylneuraminic acid synthase (Nans), cytidine monophospho‐N‐acetylneuraminic acid synthetase (Cmas), and cytidine monophospho‐N‐acetylneuraminic acid hydroxylase (Cmah), which are the key enzymes involved in the synthesis of CMP‐Neu5Gc, were significantly decreased, indicating that donor synthesis of sialic acid was blocked. What's more, a number of mannosyltransferases (Alg2, Alg8 and Alg12), sialyltransferases (st6gal1, st3gal1, st3gal3, st3gal4), and galactosyltransferases (B4galt1) were decreased.

Based on the above findings, we showed several pathways in GO analysis with significant changes about N‐glycans biosynthesis (Figure 6b,c). It is found that in Molecular Function (MF), the activities of hexose transferase were down‐regulated, and the activities of UDP‐glycosyltransferase and sialyltransferase were also affected (Figure 6c). Secondly, the cellular component (CC) was found to be down‐regulated in a variety of cellular sites enriched with glycosylation modifications (mainly endoplasmic reticulum and Golgi). However, increased synthesis of acetyl‐CoA and increased degradation of long‐chain fatty acyl‐CoA were observed. This change may be related to the rise in acetylated sialic acid (Figure 6b). According to GSEA enrichment analysis (Figure 6d), mannose transferase activity (GO:0000030) and N‐glycan biosynthesis (MMU00510) were remarkably declined after CR treatment, and many related genes were significantly affected.

## 4 DISCUSSION

We combined the serum N‐glycome and liver transcriptome to reveal changes in serum N‐glycans and associated molecular regulation basis. To our knowledge, this is the first attempt to assess the complete repertoire of serum N‐glycans level in a calorie restricted mouse model using the Bionic Glycome method by MALDI‐TOF‐MS. To detect N‐glycans from mice with long‐term CR, we collected blood samples at 7 time points and performed sequential analysis. In this study, multiple time points were observed which can help avoiding the possible bias caused by observing fewer time points and improve the reliability of the results. Meanwhile, it gave a more comprehensive picture of the changes at different ages after CR. What's more, the serum at seven time points came from the same mouse to reduce experiment variables. Fifty‐eight glycans were identified while retaining the sialic acid residues on the sialoglycans. In particular, α2,3 and α2,6‐linked sialylated glycan isomers and acetylated sialylated glycans, which have been reported to play an important role in biological processes (Bull et al., 2014; Visser et al., 2021), were also detected and quantified in this study. The global analysis of serum glycans, provides deeper insights into the molecular basis for the beneficial effects of CR on health.

We found that the glycans decreased steadily during CR treatment, and the protein concentration was not considerably impacted. Compared with the previous study of N‐glycans in CR mice by DSA‐FACE (Vanhooren et al., 2011), the content of serum N‐glycome can be relatively quantified based on the Bionic Glycome internal standard method. What's more, more fucosylated and afucosylated glycans were identified, and we also described the changes of fucosylated glycans containing sialic acid. Since we performed CR in mice of different genders, we found reduced levels of focusylation in females while elevated levels in males. These findings facilitate a more comprehensive understanding of the relationship between glycans and CR.

It is well known that majority of the proteins in serum, except immunoglobulins, are secreted by the liver. We observed the down‐regulation of N‐glycan biosynthesis through hepatic transcriptome, which provided molecular basis for the glycan changes in serum. In tumors, cancer‐specific N‐glycan alterations were reported that include high mannose accumulation caused by early synthesis cessation and upregulation of complex glycans. High expression of glycans, particularly complex multi‐antenna glycans, may encourage lymphatic metastasis and cell invasion (Oliveira‐Ferrer et al., 2017; Zhang et al., 2014). For example, compared with primary tumors in breast cancer, increased *Phaseolus vulgaris leucoagglutinin* (PHA‐L) binding in metastases and shorter survival in PHA‐L‐positive patients suggest that increasing of complex β1, 6‐branched N‐glycans contribute to breast cancer progression (Handerson et al., 2005). The accumulation of sialoglycans on the surface of various tumor cells enhances malignant features including cell migration and anti‐apoptosis (Marciel et al., 2023). Additionally, Han et al. (2020) showed that N‐glycans in mice steadily rose with age, indicating that one strategy for CR to reverse aging and improve the disease state may be by lowering the level of serum N‐glycans.

As mentioned above, the method we used was able to distinguish sialic acid glycans with different link types, and it was found that different types of sialic acid decreased. Previous studies have shown that total sialylation levels in serum appear to increase with various malignancies, and measurements of related enzymes, such as sialidase and sialyltransferase, can be used to assess cancer progression and improve prognosis in cancer patients (Zhang et al., 2018). Sialylation is characteristic of many complex glycans. Increased sialylation has been shown to improve the stability and activity of target proteins in various cancers (Vajaria et al., 2016). In addition, it is reported that upregulation of ST6GAL1 led to metastasis and spread of human colorectal cancer (CRC) cells (Park & Lee, 2013). Similarly, high ST6GAL1 expression in breast cancer cells and human anaplastic large cell lymphoma increased extracellular mechanism (ECM) adhesion and invasiveness (Suzuki et al., 2015). In aging studies, it has been found that plasma B4GALT1 and ST6GAL1 change regularly with age, and B4GALT1 keeps rising with age (Catera et al., 2016). In our research, the transcriptional levels of important enzymes (Nans, Cmas, and Cmah) in the pathway for sialic acid synthesis were dramatically reduced, indicating that sialic acid synthesis may be hampered and that low levels of sialic acid may be linked to maintaining health status.

However, there was a surprising rise in the acetylated sialic acid glycans and a stronger correlation with α2,6‐linked glycans. Our findings were further corroborated by earlier research on O‐acetylated sialic acid glycans in rat serum (Kinoshita et al., 2020). The acetylation acts as a masking agent by creating a steric barrier that prevents sialic acid proteins from interacting with other molecules (such as receptors). For example, increased O‐acetylation interferes with the Siglec family's recognition of sialic acids. When the binding of GD3 and Siglec‐7 is affected, its inhibitory effect on NK cells cytotoxicity will be destroyed (Visser et al., 2021). Additionally, it was also found that the 9‐O‐acetylation inhibited the recognition of sialic acid on receptors by influenza A and B virus hemagglutinin (Higa et al., 1985). As a result, the elevation of acetylated sialic acid in serum may hinder the normal function of sialic acid glycans, consistent with the effect by decreasing of sialic acid.

Moreover, we found that the source of acetyl‐CoA, donor of O‐acetylation, was specifically increased in the liver, which had also been found in previous studies (Mezhnina et al., 2020). Acetyl‐CoA is a central metabolite of cellular energy production in eukaryon and can be used as a donor of acetyl groups for epigenetic and post‐translational modifications (Mirzaei & Longo, 2014). The accumulation of energy‐possessing acetyl‐CoA in the energy‐deficient CR model was unexpected and, taken together with our findings, may correlate with the rise in acetylated sialic acid in the Golgi. Furthermore, we observed that the acetylated sialic acid content increased more significantly in the α2,6‐linked sialic acid, and the previous research proved our idea. For instance, SIAE expression regulates sialic acid levels in cells. A sharp increase in α2,6‐linked sialic acid and a slight increase in α2,3‐linked sialic acid were observed in HCT 116 cell lines with SIAE knockdown (Grabenstein et al., 2021). In addition, a highly selective loss of 9‐O‐acetylation of sialic acid was observed in ST6Gal1 knockout mice (Grabenstein et al., 2021; Martin et al., 2002). Therefore, O‐acetylation is substantially correlated with ST6Gal1‐mediated α2,6 linkage, which may be more common on α2,6‐linked sialic acids.

In conclusion, we observed a general reduction in the serum N‐glycome and provided a molecular basis for changes of N‐glycans through gene regulation at the liver transcriptome level. Although the molecular mechanism of how calorie restriction affects N‐glycan biosynthesis remains to be explored, our finding provides the potential biomarker for monitoring and intervention of aging and age‐related diseases.

## 5 CONCLUSION

We revealed the changes of serum N‐glycans and gene expression differences in C57BL/6 mice under calorie restriction model by combining glycome and transcriptome. The results showed that the level of N‐glycans, except for that of O‐acetylated sialic acid glycans, decreased significantly in response to calorie restriction. We used RNA‐seq to reveal the transcriptome change pattern of liver under CR, and observed down‐regulation of N‐glycan synthesis pathway and upregulation of acetyl‐CoA synthesis related pathway. Given the masking effect of O‐acetylation on terminal sialic acid, we hypothesized that low levels of serum N‐glycans might be associated with health status under calorie restriction. This study provides a basis for exploring the function of N‐glycans and the mechanism of caloric restriction.

## AUTHOR CONTRIBUTIONS

**Jiteng Fan:** Experiment design, mouse breeding, experiment implementation, data processing, writing‐original draft, writing review and editing. **Jichen Sha:** Mouse breeding, blood sampling, writing review and editing. **Shuwai Chang:** Mouse breeding, experiment implementation, writing review and editing. **Huijuan Zhao**: Mouse breeding, tissue collecting, writing review and editing. **Xiaoyun Niu:** Tissue collecting, writing review and editing. **Yong Gu:** Blood sampling and writing review and editing. **Shifang Ren:** Experiment design, writing review and editing. **Jianxin Gu:** Experiment design, writing review and editing.

## CONFLICT OF INTEREST STATEMENT

The authors declare no conflict of interest.

## Supporting information


Figure S1.

Table S1–S4.

## Data Availability

All data presented in this study are available upon request. Please contact the corresponding author for details.
