# Supplementary material for: Generalized low levels of serum N‐glycans associate with better health status
Source: Aging Cell. 2023 May 2;22(7):e13855. doi: 10.1111/acel.13855 (PMC10352567; doi:10.1111/acel.13855)
Supplement: Supplementary file 1 — Figure S1. [file ACEL-22-e13855-s002.pdf]

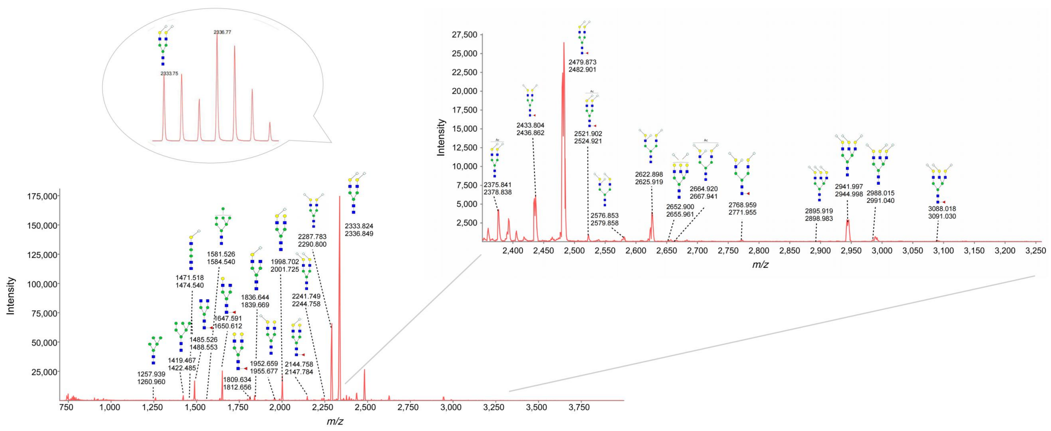

Figure S1 The representative MALDI-TOF-MS spectrum of serum N-glycome, based on Bionic Glycome Method (only partial glycans are labeled to ensure a clear picture). A total 58 pairs of glycans with $[M + Na]^+$ were detected.

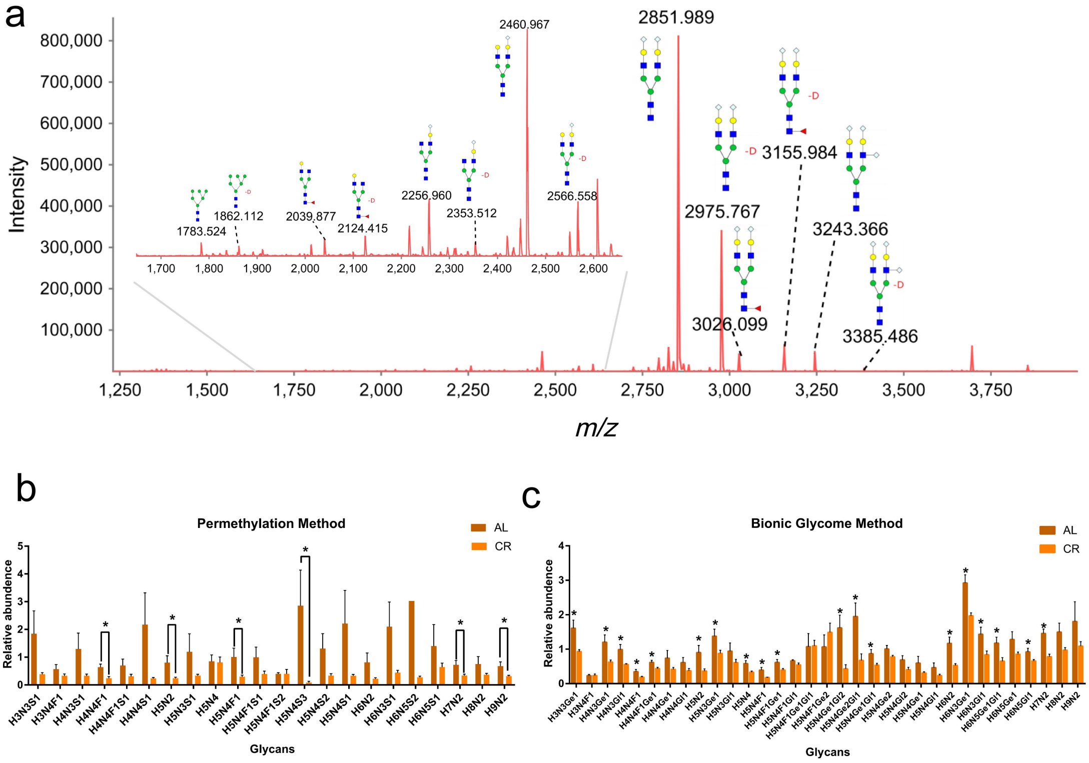

Figure S2 Comparison of isotope-labeled permethylation internal standard quantification method and Bionic Glycome Method. (a) Representative mass spectra of permethylation quantification method. The red "-D" represents the deuterated methylated labeled glycans. (b-c) The glycans identified by both permethylation method and Bionic Glycome Method (n[AL]=4, n[CR]=5). Data are presented as mean ± SEM. The p-value was considered significant if it was below 0.05 and $*p < 0.05$.

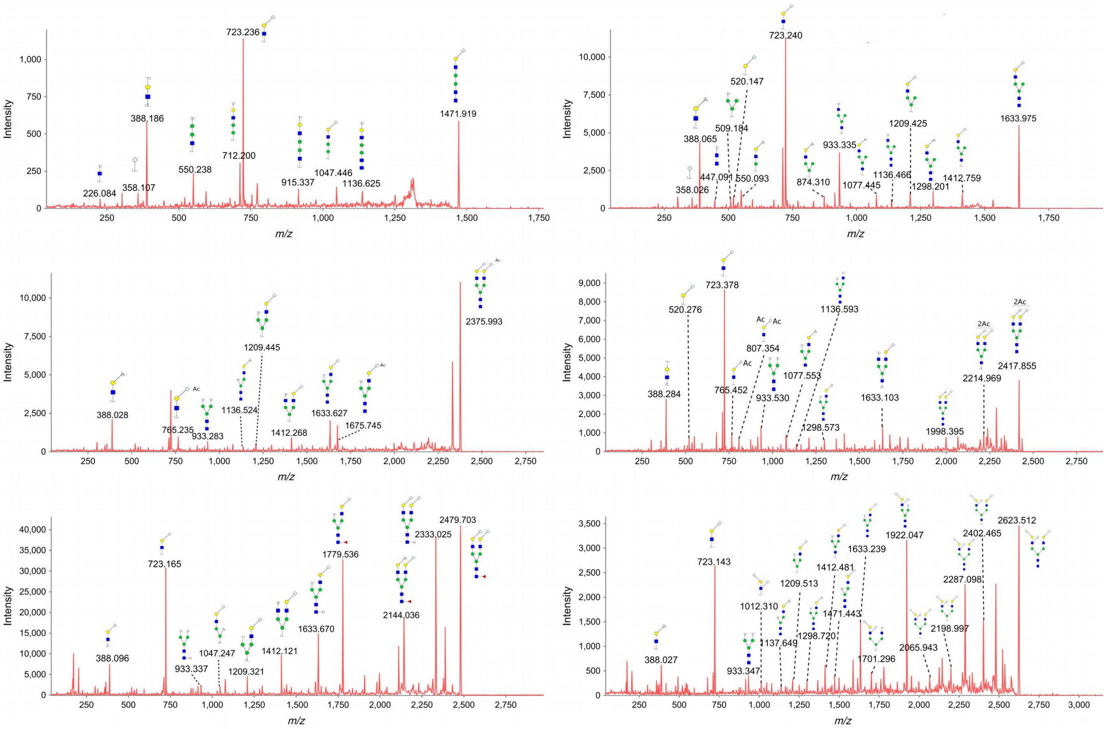

Figure S3 Spectrum of tandem mass spectrometry of partial N-glycans found in mouse serum.

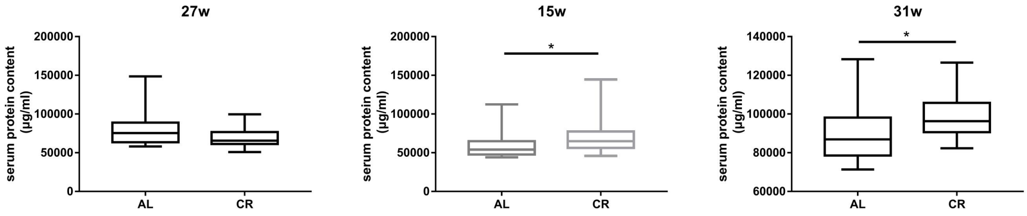

Figure S4 The protein content in serum at three time points (15w, 27w, 31w) between AL and CR group. The p-value was considered significant if it was below 0.05 and *$p < 0.05$.

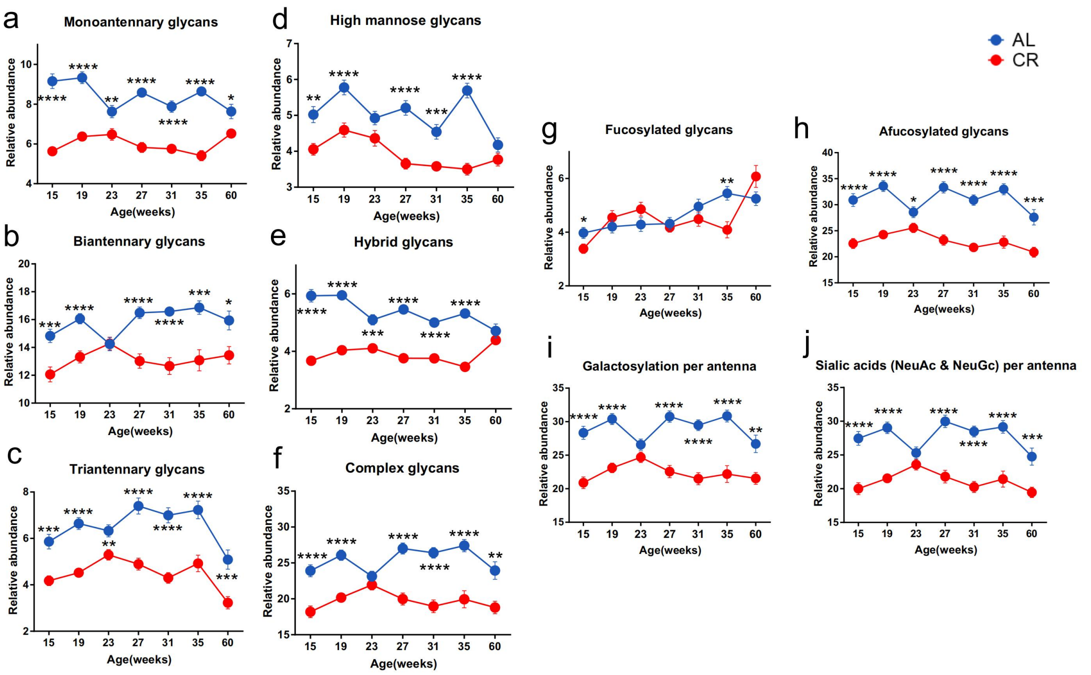

Figure S5 The dynamic changes with age of derived glycosylation traits in AL and CR group. (a-c) Monoantennary, biantennary and triantennary glycans were distinguished by antenna numbers. (d-f) As for structure, derived features included high mannose glycans, hybrid glycans and complex glycans. (g-j) According to the monosaccharides of glycans, four traits were divided into fucosylation, afucosylation, galactosylation per antenna, and sialylation per antenna. Data are presented as mean ± SEM. The difference between groups at each point were compared using a two-tailed t test. The p-value was considered significant if it was below 0.05. *$p < 0.05$; **$p < 0.01$; ***$p < 0.001$; ****$p < 0.0001$.

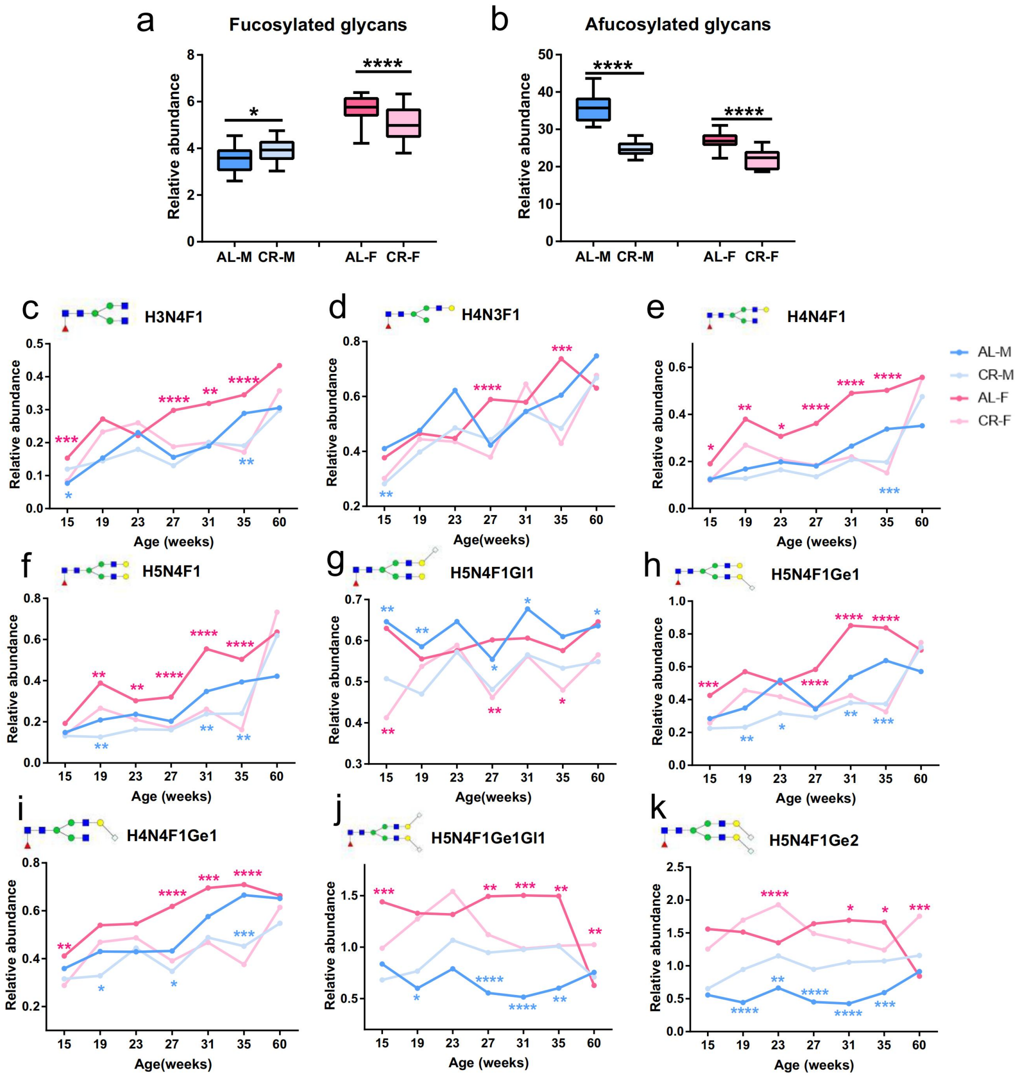

Figure S6 Fucosylation level changes in AL and CR groups. (a-b) The difference of fucosylation and afucosylation in different genders and different groups of mice. (c-k) Changes of 9 fucosylated glycans during calorie restriction in AL and CR groups of different genders. AL-M and CR-M: male mice in AL and CR group; AL-F and CR-F: female mice in AL and CR group.

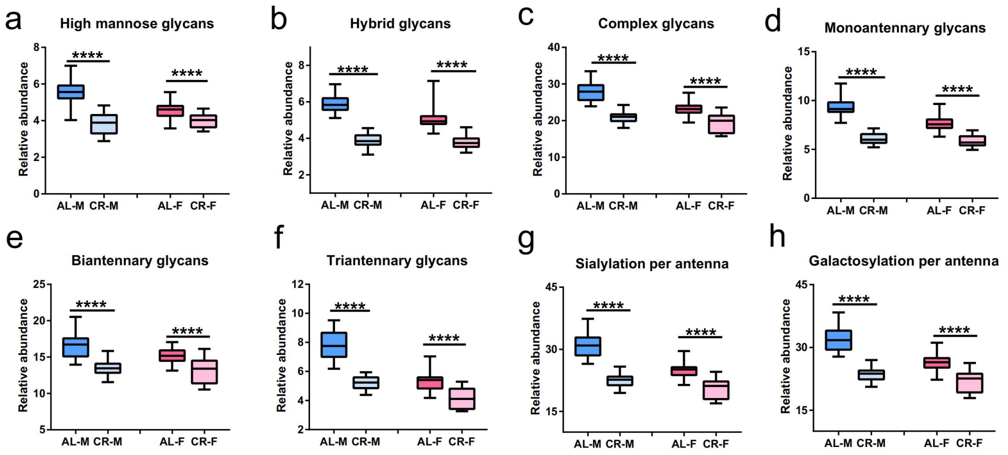

Figure S7 The relative abundance of derived glycosylation traits in male and female between AL and CR group. AL-M and CR-M: male mice in AL and CR group; AL-F and CR-F: female mice in AL and CR group.

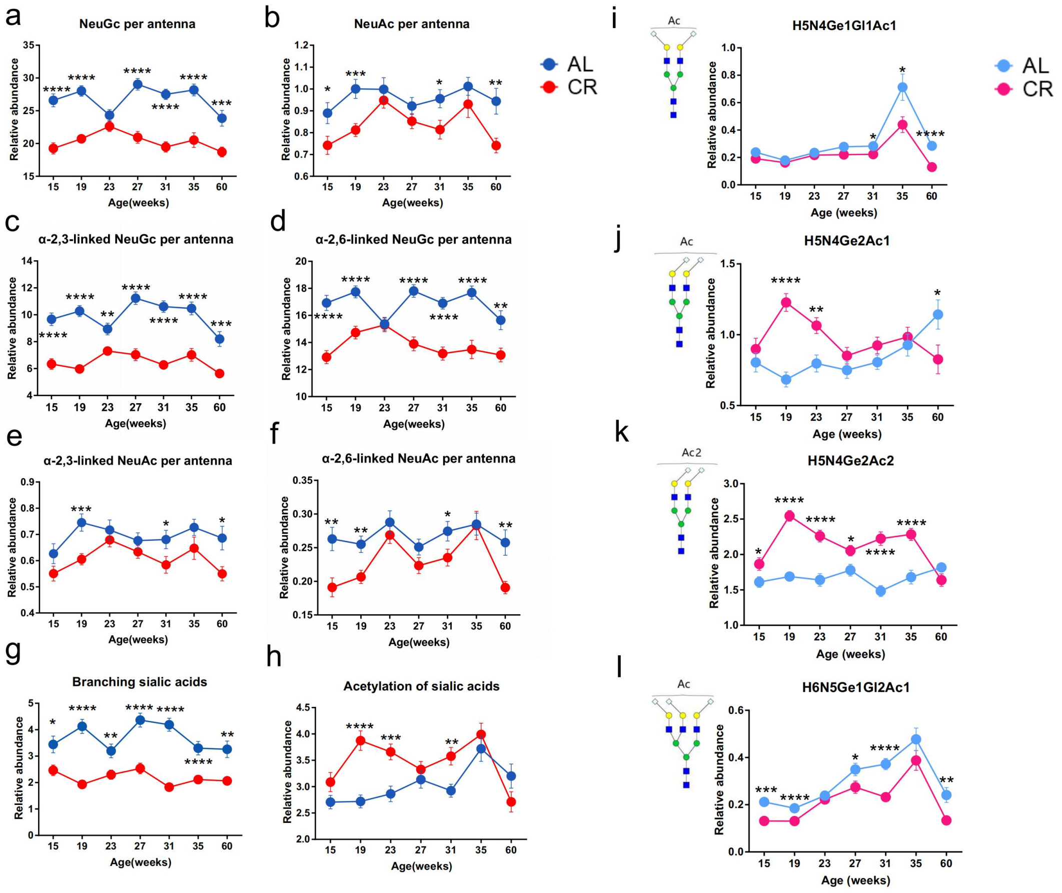

Figure S8 The dynamic changes with age in derived traits of sialylation and O-acetylated sialic acid glycans between AL and CR group. (a-h) Based on the link types and modification signatures, a total of 8 derived traits of sialylation were divided. “Branched sialic acid” is the glycans with disialylated antennae. (i-l) Changes of the glycans with O-acetylated sialic acid during CR. Data are presented as mean ± SEM. The difference at each point were compared using a two-tailed t test. The p-value was considered significant if it was below 0.05. *$p < 0.05$; **$p < 0.01$; ***$p < 0.001$; ****$p < 0.0001$.

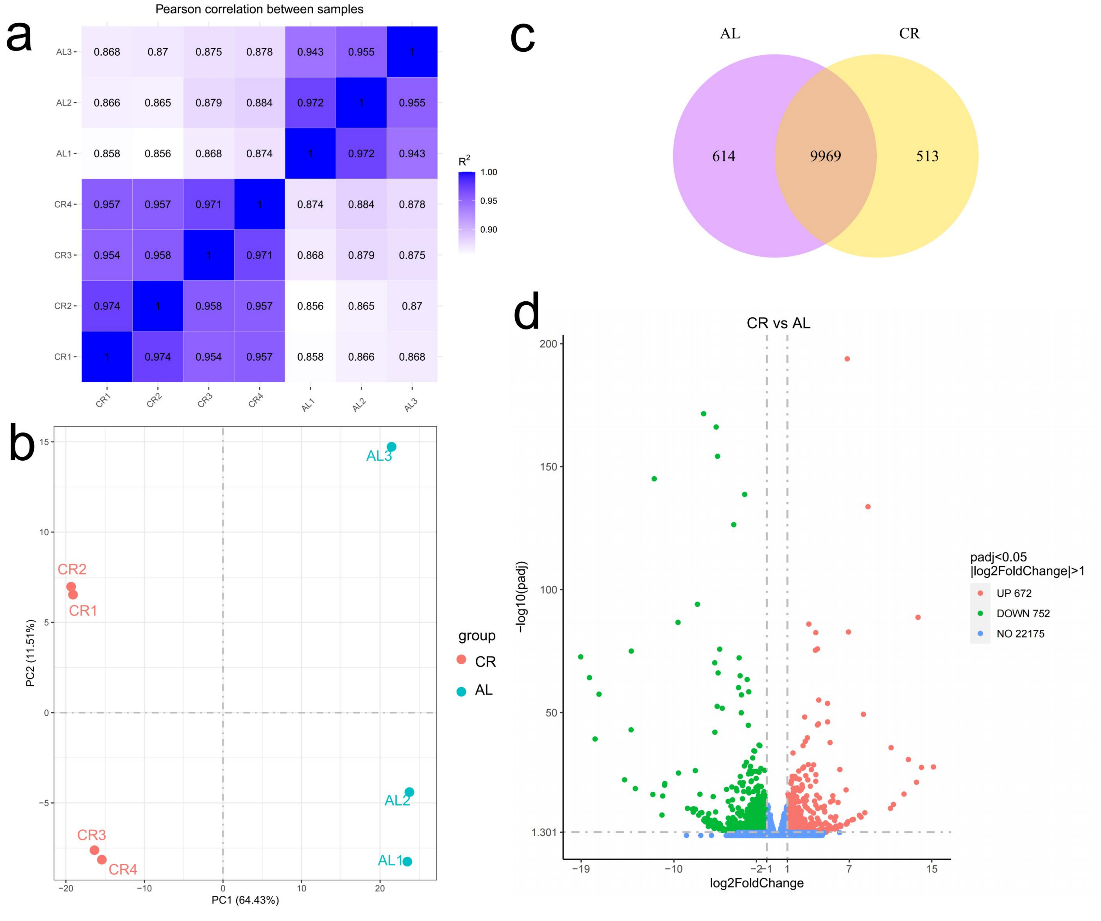

Figure S9 The transcriptome analysis in liver of 30-week-old male mice. (a) Pearson correlation coefficient in AL and CR samples. (b) Principal component analysis (PCA) of the liver transcriptome was performed in mice between different feeding patterns. (c) Venn diagram showed the differential expression of AL and CR groups. (d) Volcano plot showed up-regulated and down-regulated genes in CR vs AL.

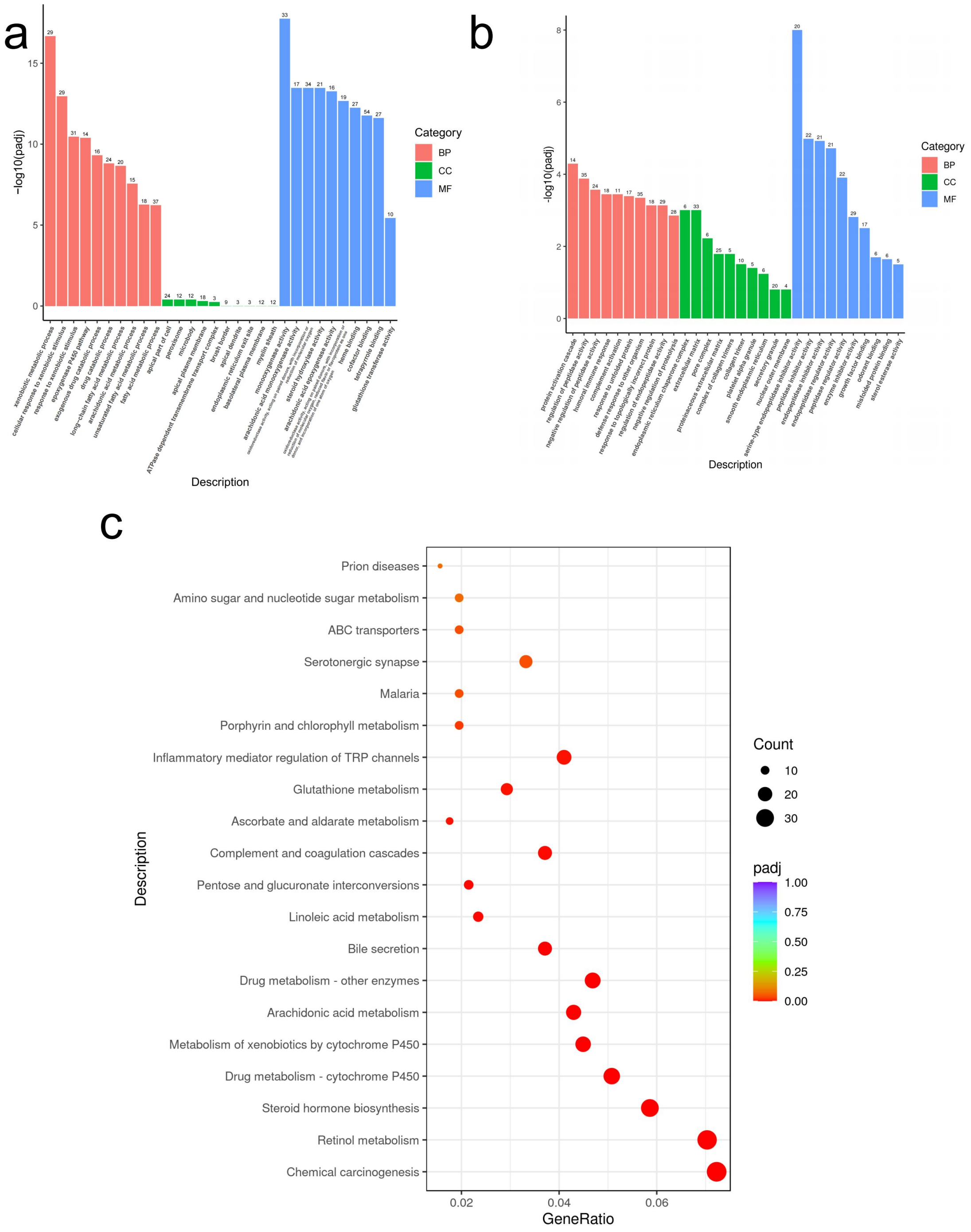

Figure S10 The enrichment analysis based on RNA-seq in GO and KEGG. (a-b) The top 10 enrichment in Biological Process (BP), Cellular Component (CC), and Molecular Function (MF) of up-regulated and down-regulated in GO analysis. (c) The top 20 enriched pathways in KEGG pathway analysis.
